# Supplementary material for: Misclassification of yellow fever vaccination status revealed through hierarchical Bayesian modeling
Source: Am J Epidemiol. 2025 Mar 21;194(10):2879–86. doi: 10.1093/aje/kwae465 (PMC12527270; doi:10.1093/aje/kwae465)
Supplement: Web_Material_kwae465 [file web_material_kwae465.pdf]

Supplement: Misclassification of yellow fever vaccination status revealed through hierarchical Bayesian modeling

List of Figures

S1 **Relationship between the number of data points from each country (y-axis) and uncertainty in the estimates of Se_{vac} (left) and Sp_{vac} (right) for the default model**. Points and lines represent the posterior medians and 95% credible intervals of Se_{vac} and Sp_{vac} for each country. 4

S2 **Estimates of Se_{vac} and Sp_{vac} from the default model after excluding the top five countries with the most data points**. The dots and lines represent posterior medians and 95% credible intervals of country-specific estimates of $Se_{vac,k}$ and $Sp_{vac,k}$. The orange dots and lines indicate the top five countries with the most data points. Lighter shading indicates higher probability densities. 5

S3 **Estimates of Se_{vac} (y-axis) and Sp_{vac} (x-axis) from the 16 models (panels)**. In the columns and rows, geographic variability is indicated by whether parameters vary by country or do not vary and are thus global constants. The default model (top left) includes geographic variability in Se_{vac} and Sp_{vac} (vac), Se_{test} and Sp_{test} (test), force of infection (FOI), and reporting proportion (rep). The dots and lines represent posterior medians and 95% credible-intervals of country-specific estimates of $Se_{vac,k}$ and $Sp_{vac,k}$. Lighter shading indicates higher probability densities. 6

S4 **Marginal posterior distributions of Se_{vac} (blue) and Sp_{vac} (red) from the eight models (panels) that accounted for geographic variability in those parameters**. The dots and lines represent medians and 95% credible intervals of country-specific estimates of $Se_{vac,k}$ and $Sp_{vac,k}$. The country-specific estimates are sorted in ascending order based on $Se_{vac,k}$. A noticeable trend of negative correlation between $Se_{vac,k}$ and $Sp_{vac,k}$ is observed for all models. 7

S5 **Estimation of $Se_{vac,k}$ (x-axis) and $Sp_{vac,k}$ (y-axis) for each of 20 countries (panels) from ten data sets simulated with posterior median parameter values.** The ten black dots in each panel and their lines represent median estimates and 95% credible intervals obtained from our analysis of the ten simulated data sets. The red dots and gray areas represent median posterior estimates and their corresponding 95% credible intervals obtained from our analysis of empirical data. The red dots were also the parameter values used to generate the ten simulated data sets. 8

S6 **Estimates of Se_{test} and Sp_{test} from the default model**. The points with their lines represent the median estimates and the 95% CrI of country-specific estimates $Se_{test,k}$ and $Sp_{test,k}$. The red points and lines indicate the medians and 95% uncertainty intervals of the priors used. 9

S7 **Estimates of Se_{test} (y-axis) and Sp_{test} (x-axis) from the 16 models (panels)**. In the columns and rows, geographic variability is indicated by whether parameters vary by country or do not vary and are thus global constants. The default model (top left) includes geographic variability in Se_{vac} and Sp_{vac} (vac), Se_{test} and Sp_{test} (test), force of infection (FOI), and reporting proportion (rep). The dots and lines represent posterior median estimates and the 95% credible intervals of country-specific estimates $Se_{test,k}$ and $Sp_{test,k}$. The red points and lines indicate the medians and 95% uncertainty intervals of the priors. 10

S8 **Estimates of force of infection, λ_k, (y-axis) from the 16 models (panels)**. Dots and lines represent posterior medians and 95% credible intervals of λ_k for each country. Vertical red lines represent the 95% intervals of the prior distributions, which were obtained from a previous study [1]. The default model (top left) includes geographic variability in Se_{vac} and Sp_{vac} (vac), Se_{test} and Sp_{test} (test), force of infection (FOI), and reporting proportion (rep). In the columns and rows, geographic variability is indicated by whether parameters vary by country or do not vary and are thus global constants. . 11
S9 **Estimates of reporting proportion, ρ_k, from the 16 models (panels)**. Black dots and lines represent posterior medians and 95% credible intervals of ρ_k for each country (or for the global scale, depending on the model). Red lines represent the 95% interval and the horizontal black lines represent the median of the prior distribution, which was obtained from a previous study [1]. The default model (top left) includes geographic variability in Se_{vac} and Sp_{vac} (vac), Se_{test} and Sp_{test} (test), force of infection (FOI), and reporting proportion (rep). In the columns and rows, geographic variability is indicated by whether parameters vary by country or do not vary and are thus global constants. 12
S10 **Estimates of vaccine efficacy, VE, (y-axis) from all 16 models (x-axis)**. Black dots and lines depict posterior medians and 95% credible intervals for VE. The horizontal red lines represent the 95% interval of the prior distribution, and the black line represents the median of the prior distribution. 13
S11 **Estimates of $Se_{vac,k}$ (left) and $Sp_{vac,k}$ (right) from simulated data with (y-axis) or without (x-axis) changed values of λ_k for each of 20 countries (panels).** In the unchanged scenario, we simulated ten data sets with posterior median parameter values. In the changed scenario, we simulated ten data sets with λ_k set to values of either 10^{-5}, 10^{-4}, 10^{-3}, 10^{-2}, or 10^{-1}. The ten black dots in each panel and their lines represent median estimates and 95% credible intervals obtained from our analysis of the ten simulated data sets. The red dots and gray areas represent median posterior estimates and their corresponding 95% credible intervals obtained from our analysis of empirical data. 14
S12 **Estimates of force of infection, λ_k, (y-axis) for each of 20 countries (x-axis) in ten simulated data sets (panels).** Black dots and vertical lines denote posterior median and 95% credible intervals. Vertical red lines represent the 95% intervals of the prior distributions, sourced from a previous study [1]. Triangles correspond to the values of λ_k used to generate the simulated data. 15
S13 **Estimates of $Se_{vac,k}$ (left) and $Sp_{vac,k}$ (right) from simulated data with (y-axis) or without (x-axis) changed values of $Se_{test,k}$ and $Sp_{vac,k}$ for each of 20 countries (panels).** In the unchanged scenario, we simulated ten data sets with posterior median parameter values. In the changed scenario, we simulated ten data sets with $Se_{test,k}$ and $Sp_{test,k}$ set to 0.7. The ten black dots in each panel and their lines represent median estimates and 95% credible intervals obtained from our analysis of the ten simulated data sets. The red dots and gray areas represent median posterior estimates and their corresponding 95% credible intervals obtained from our analysis of empirical data. 16

List of Tables

S1 **Correlation between $Se_{vac,k}$ and $Sp_{vac,k}$ across countries in eight models that account for geographic variability in Se_{vac} and Sp_{vac}.** 17

1 Figures

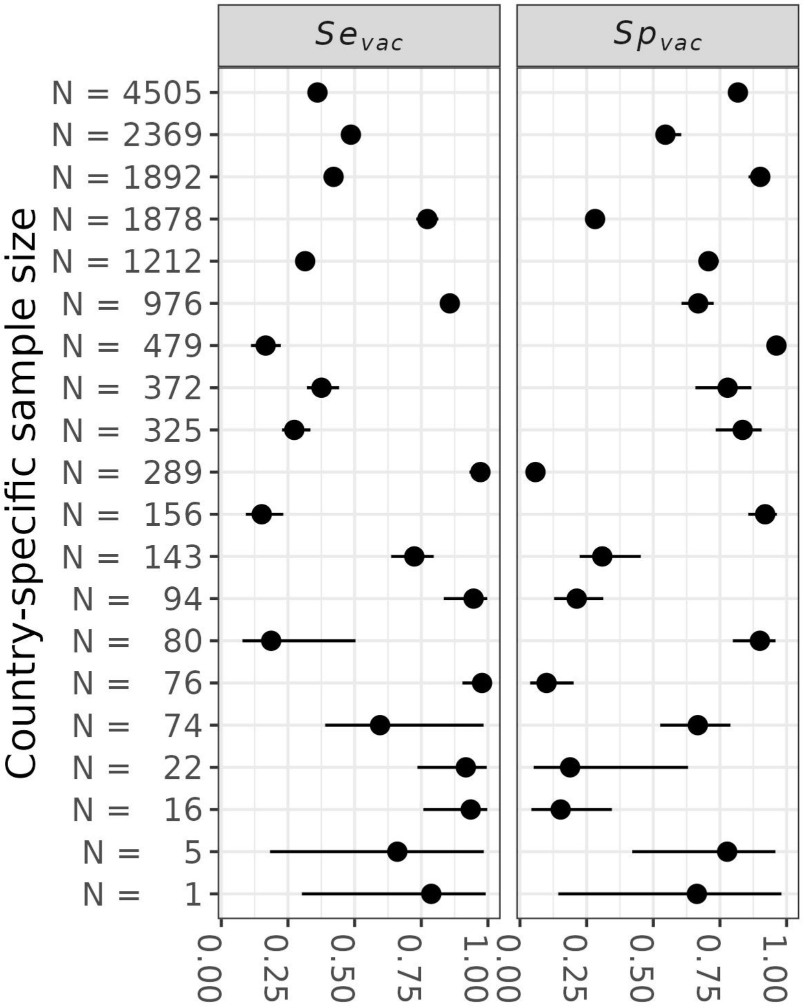

Figure S1: **Relationship between the number of data points from each country (y-axis) and uncertainty in the estimates of Se_{vac} (left) and Sp_{vac} (right) for the default model**. Points and lines represent the posterior medians and 95% credible intervals of Se_{vac} and Sp_{vac} for each country.

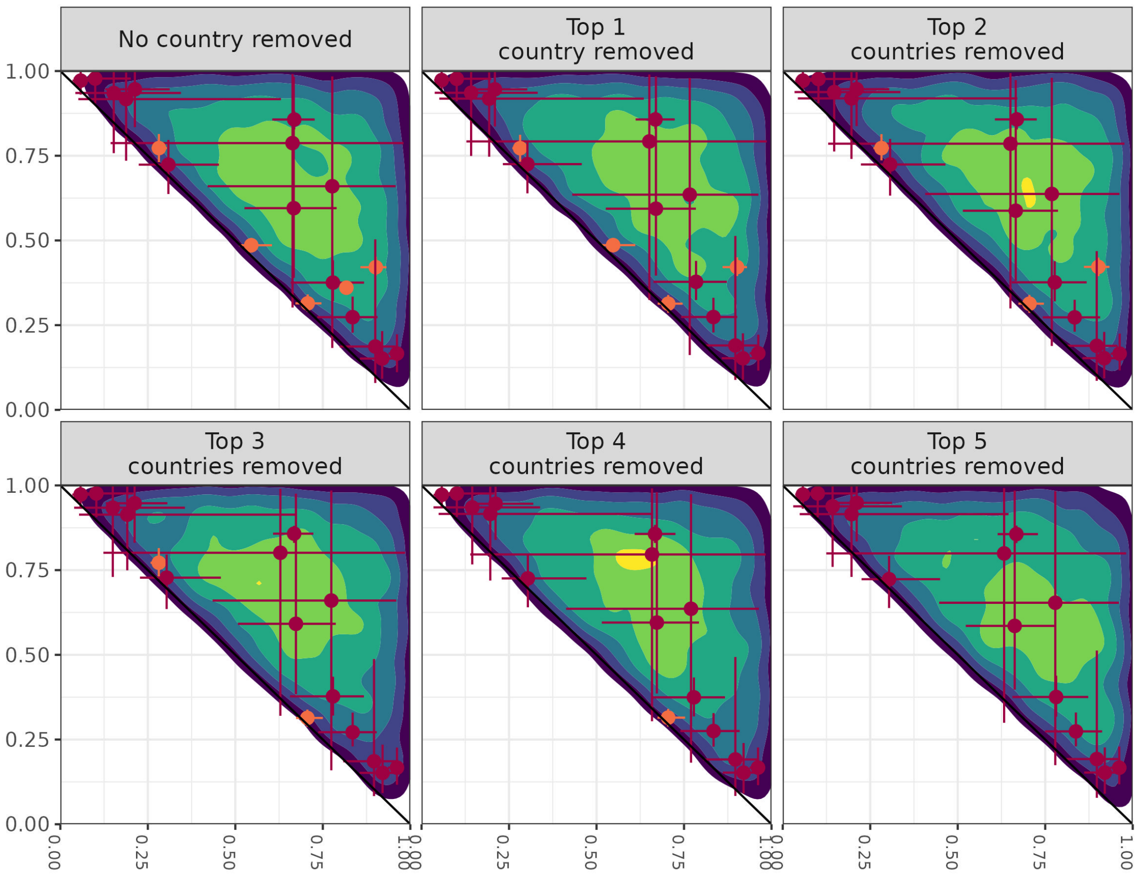

Figure S2: **Estimates of Se_{vac} and Sp_{vac} from the default model after excluding the top five countries with the most data points**. The dots and lines represent posterior medians and 95% credible intervals of country-specific estimates of $Se_{vac,k}$ and $Sp_{vac,k}$. The orange dots and lines indicate the top five countries with the most data points. Lighter shading indicates higher probability densities.

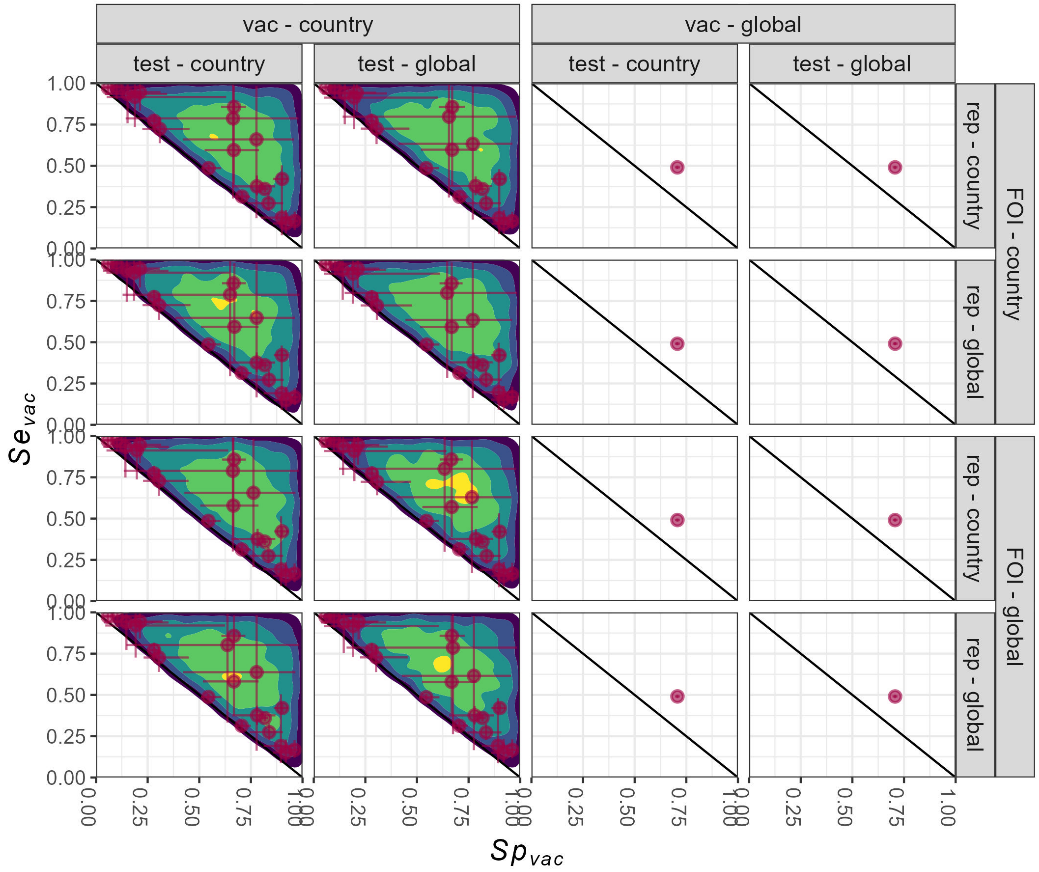

Figure S3: **Estimates of Se_{vac} (y-axis) and Sp_{vac} (x-axis) from the 16 models (panels)**. In the columns and rows, geographic variability is indicated by whether parameters vary by country or do not vary and are thus global constants. The default model (top left) includes geographic variability in Se_{vac} and Sp_{vac} (vac), Se_{test} and Sp_{test} (test), force of infection (FOI), and reporting proportion (rep). The dots and lines represent posterior medians and 95% credible-intervals of country-specific estimates of $Se_{vac,k}$ and $Sp_{vac,k}$. Lighter shading indicates higher probability densities.

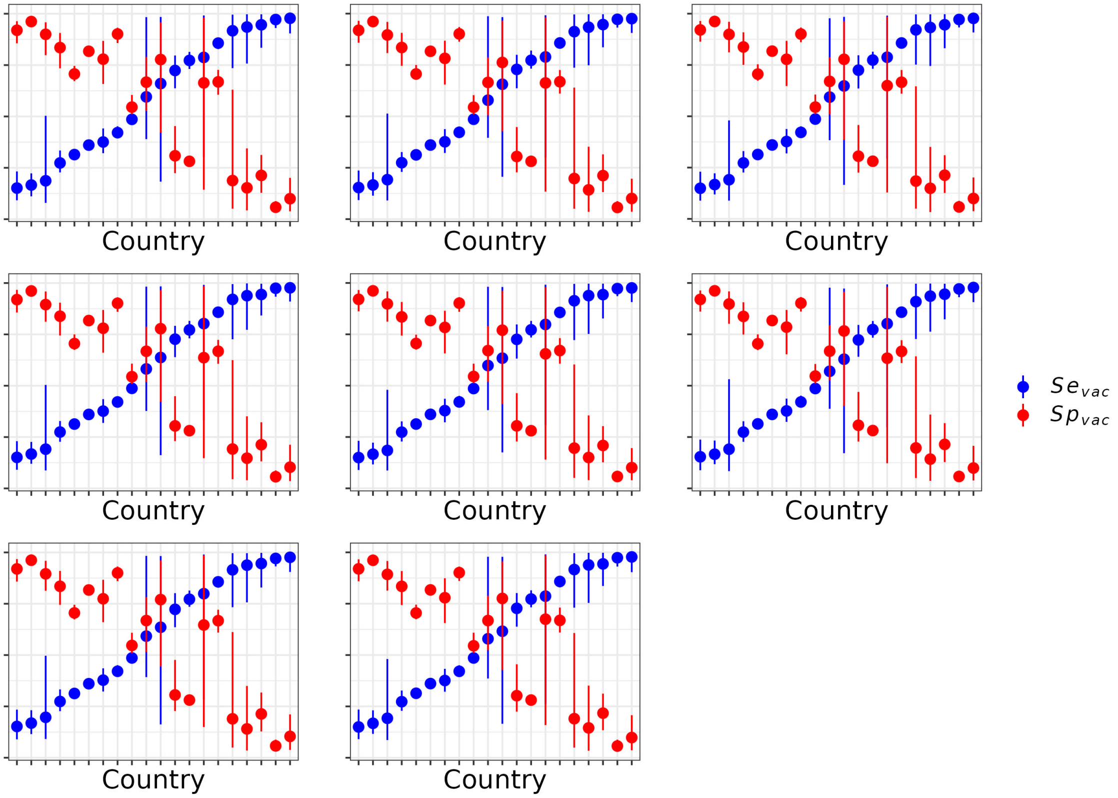

Figure S4: **Marginal posterior distributions of Se_{vac} (blue) and Sp_{vac} (red) from the eight models (panels) that accounted for geographic variability in those parameters**. The dots and lines represent medians and 95% credible intervals of country-specific estimates of $Se_{vac,k}$ and $Sp_{vac,k}$. The country-specific estimates are sorted in ascending order based on $Se_{vac,k}$. A noticeable trend of negative correlation between $Se_{vac,k}$ and $Sp_{vac,k}$ is observed for all models.

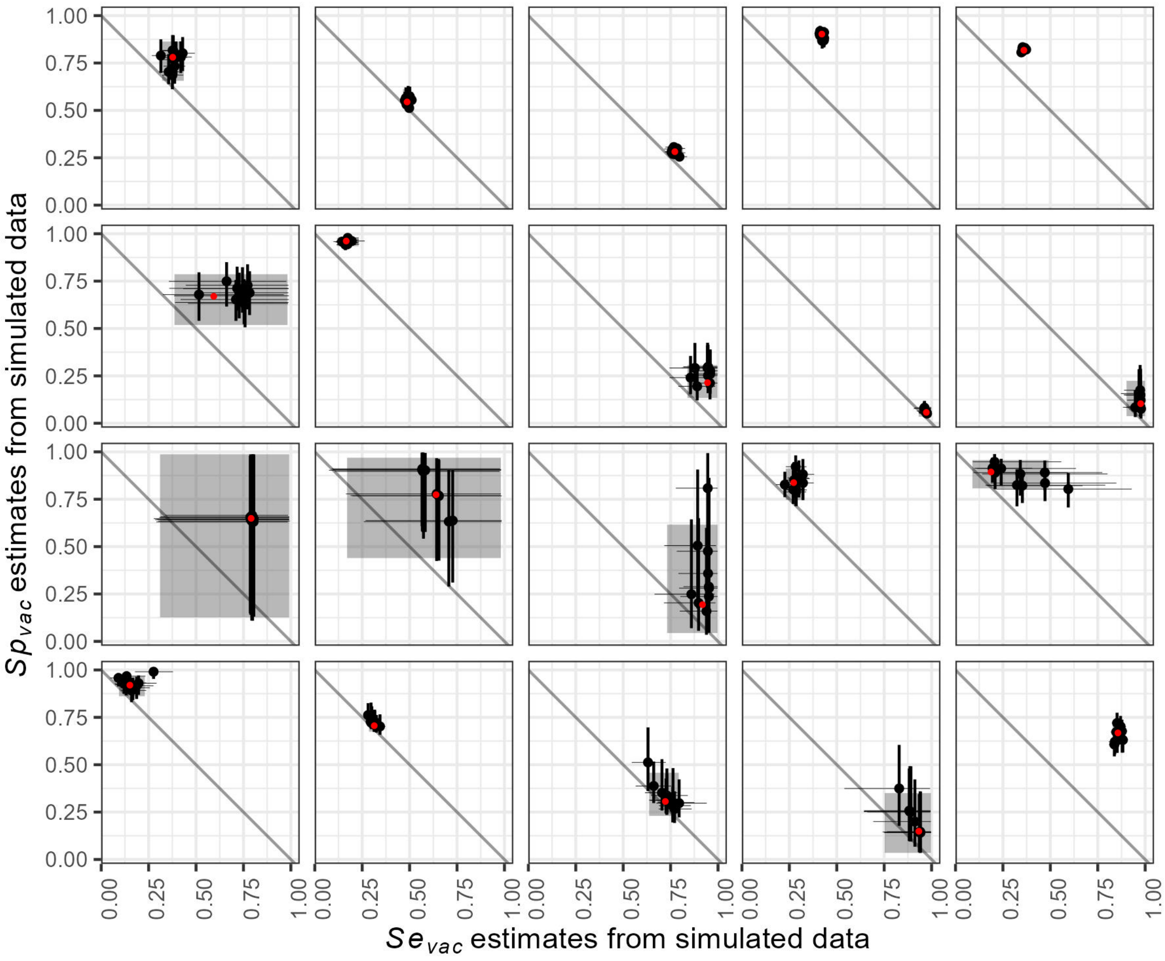

Figure S5: **Estimation of $Se_{vac,k}$ (x-axis) and $Sp_{vac,k}$ (y-axis) for each of 20 countries (panels) from ten data sets simulated with posterior median parameter values.** The ten black dots in each panel and their lines represent median estimates and 95% credible intervals obtained from our analysis of the ten simulated data sets. The red dots and gray areas represent median posterior estimates and their corresponding 95% credible intervals obtained from our analysis of empirical data. The red dots were also the parameter values used to generate the ten simulated data sets.

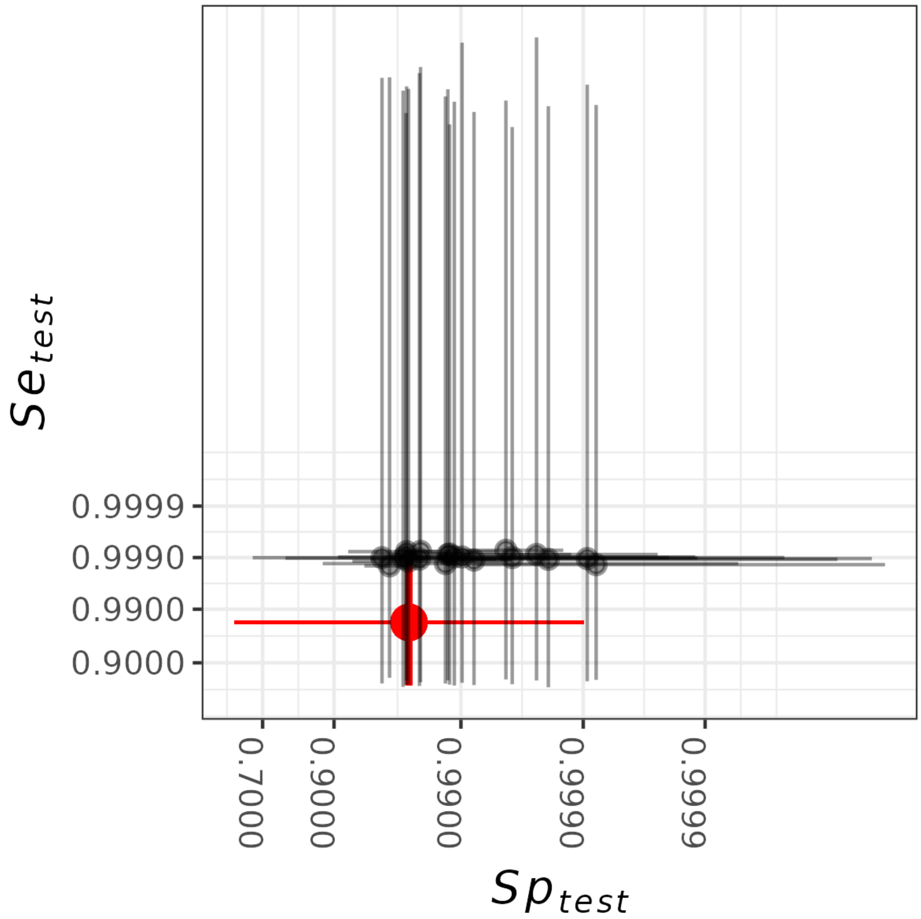

Figure S6: **Estimates of Se_{test} and Sp_{test} from the default model**. The points with their lines represent the median estimates and the 95% CrI of country-specific estimates $Se_{test,k}$ and $Sp_{test,k}$. The red points and lines indicate the medians and 95% uncertainty intervals of the priors used.

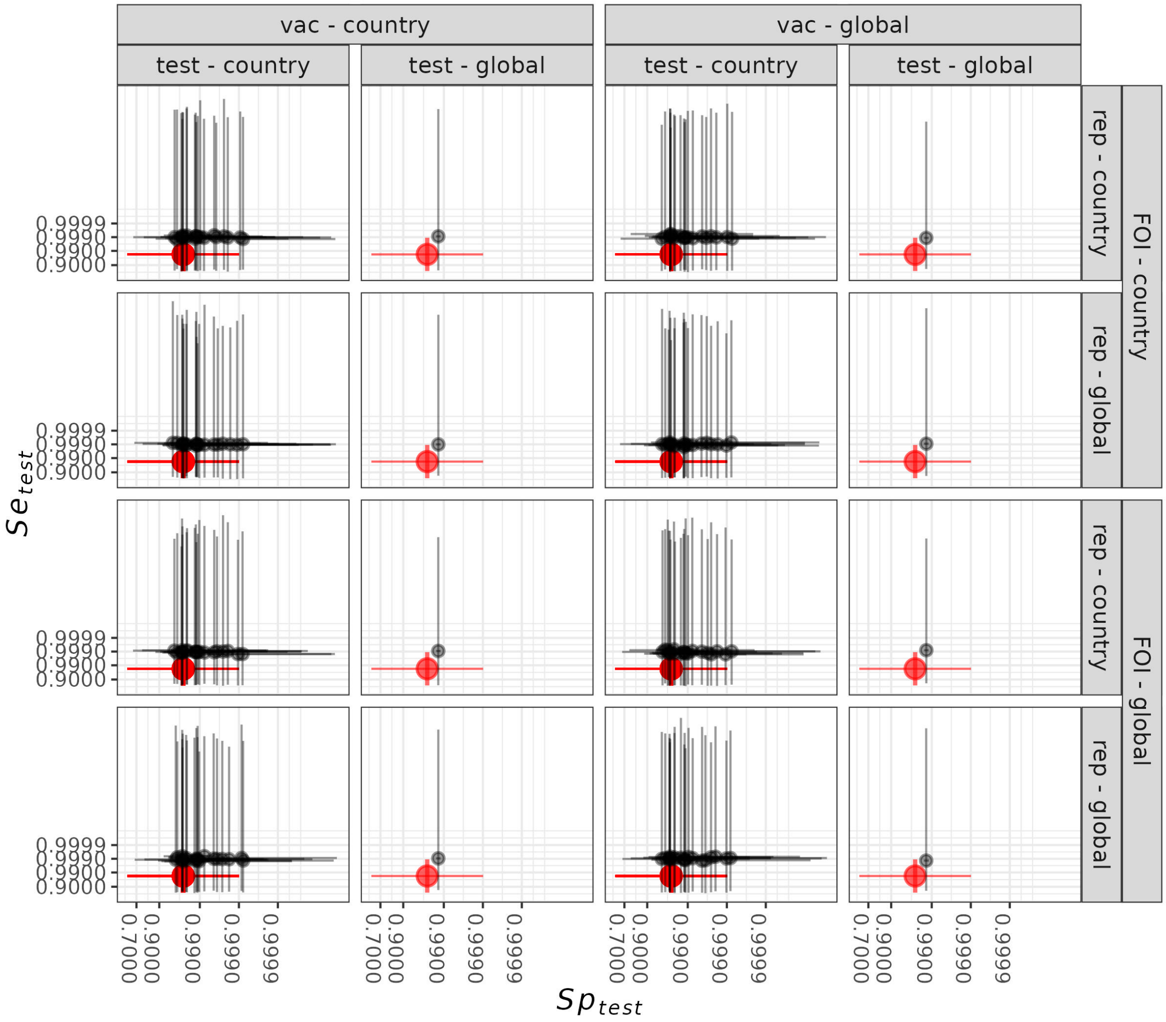

Figure S7: **Estimates of Se_{test} (y-axis) and Sp_{test} (x-axis) from the 16 models (panels)**. In the columns and rows, geographic variability is indicated by whether parameters vary by country or do not vary and are thus global constants. The default model (top left) includes geographic variability in Se_{vac} and Sp_{vac} (vac), Se_{test} and Sp_{test} (test), force of infection (FOI), and reporting proportion (rep). The dots and lines represent posterior median estimates and the 95% credible intervals of country-specific estimates $Se_{test,k}$ and $Sp_{test,k}$. The red points and lines indicate the medians and 95% uncertainty intervals of the priors.

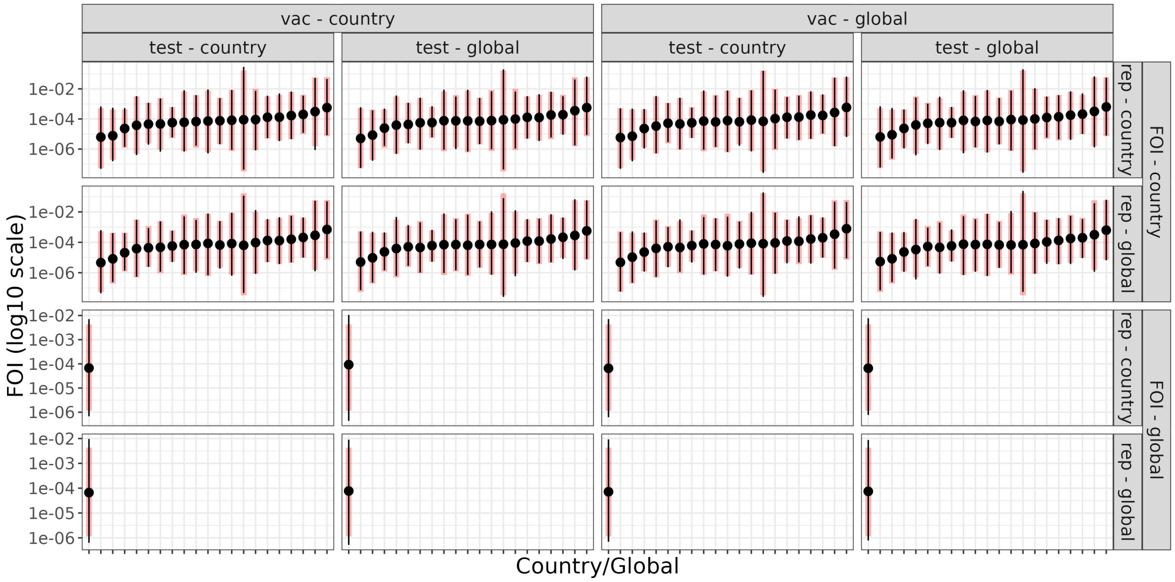

Figure S8: **Estimates of force of infection, λ_k, (y-axis) from the 16 models (panels)**. Dots and lines represent posterior medians and 95% credible intervals of λ_k for each country. Vertical red lines represent the 95% intervals of the prior distributions, which were obtained from a previous study [1]. The default model (top left) includes geographic variability in Se_{vac} and Sp_{vac} (vac), Se_{test} and Sp_{test} (test), force of infection (FOI), and reporting proportion (rep). In the columns and rows, geographic variability is indicated by whether parameters vary by country or do not vary and are thus global constants.

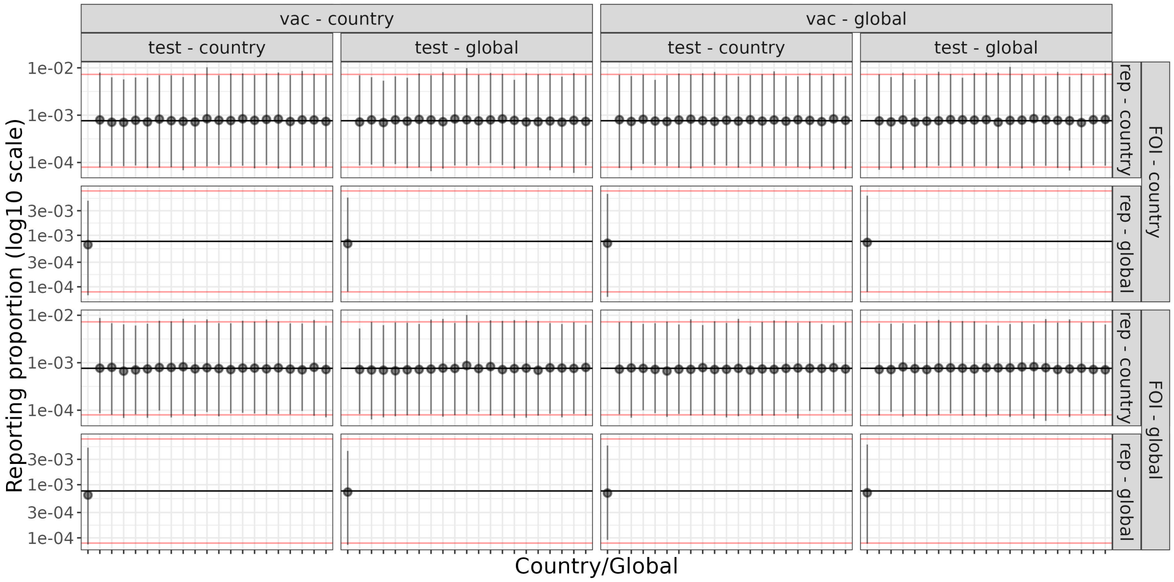

Figure S9: **Estimates of reporting proportion, ρ_k, from the 16 models (panels)**. Black dots and lines represent posterior medians and 95% credible intervals of ρ_k for each country (or for the global scale, depending on the model). Red lines represent the 95% interval and the horizontal black lines represent the median of the prior distribution, which was obtained from a previous study [1]. The default model (top left) includes geographic variability in Se_{vac} and Sp_{vac} (vac), Se_{test} and Sp_{test} (test), force of infection (FOI), and reporting proportion (rep). In the columns and rows, geographic variability is indicated by whether parameters vary by country or do not vary and are thus global constants.

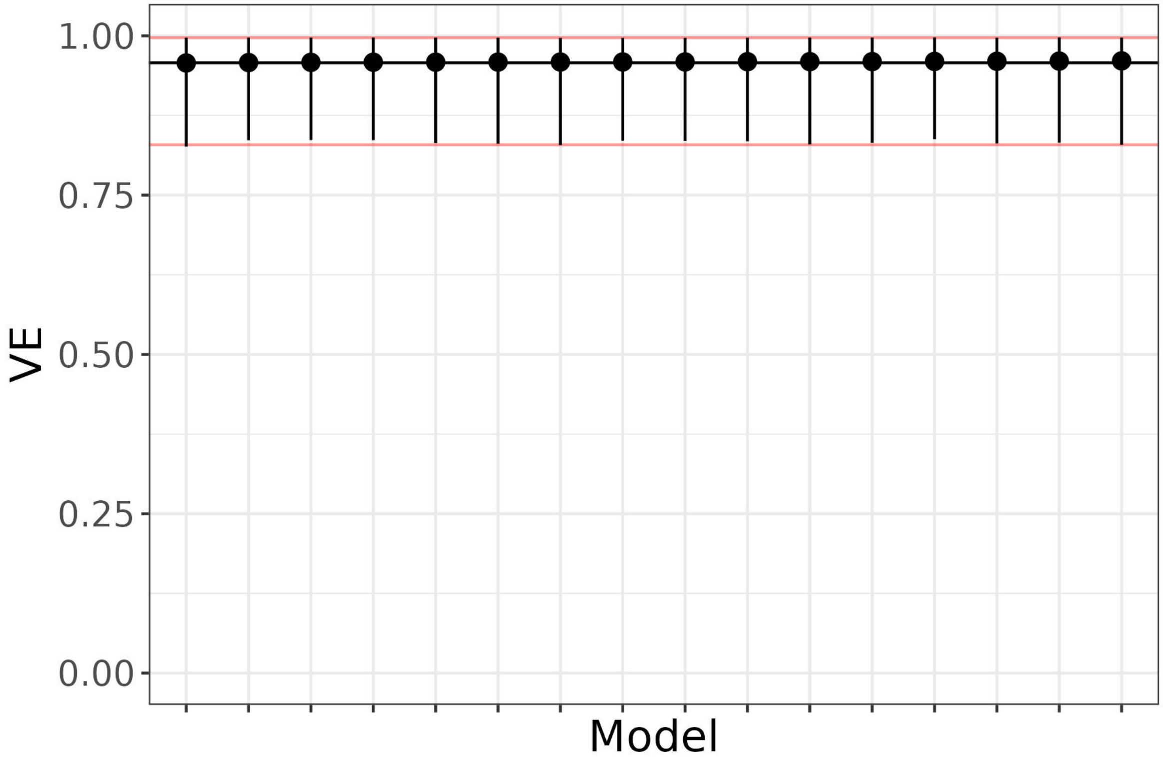

Figure S10: **Estimates of vaccine efficacy, VE, (y-axis) from all 16 models (x-axis)**. Black dots and lines depict posterior medians and 95% credible intervals for VE. The horizontal red lines represent the 95% interval of the prior distribution, and the black line represents the median of the prior distribution.

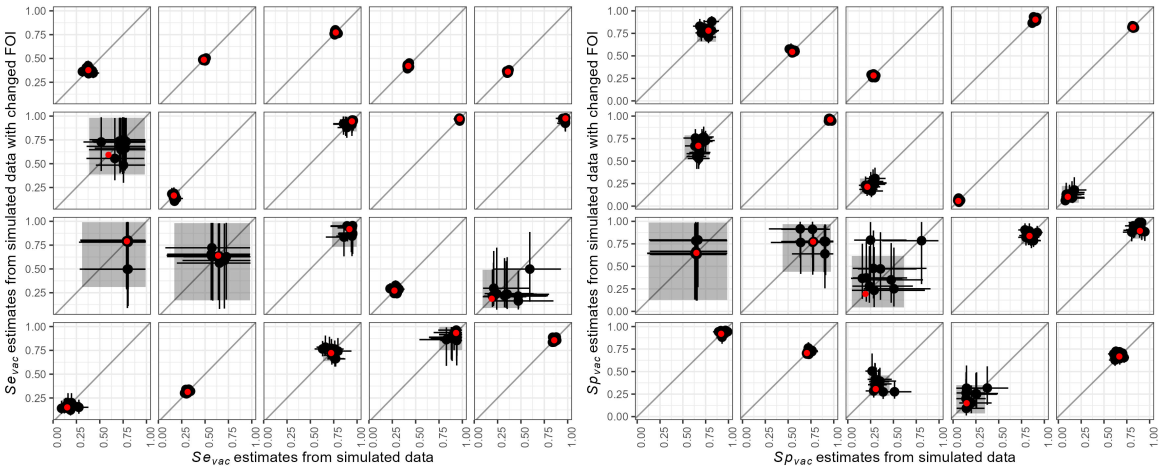

Figure S11: **Estimates of $Se_{vac,k}$ (left) and $Sp_{vac,k}$ (right) from simulated data with (y-axis) or without (x-axis) changed values of λ_k for each of 20 countries (panels).** In the unchanged scenario, we simulated ten data sets with posterior median parameter values. In the changed scenario, we simulated ten data sets with λ_k set to values of either 10^{-5}, 10^{-4}, 10^{-3}, 10^{-2}, or 10^{-1}. The ten black dots in each panel and their lines represent median estimates and 95% credible intervals obtained from our analysis of the ten simulated data sets. The red dots and gray areas represent median posterior estimates and their corresponding 95% credible intervals obtained from our analysis of empirical data.

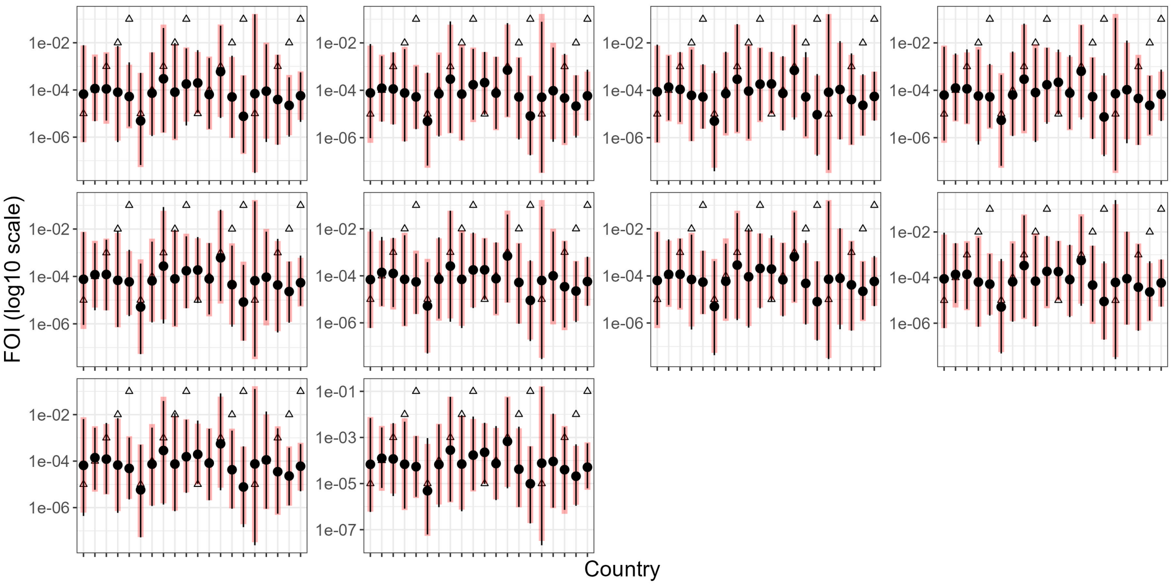

Figure S12: **Estimates of force of infection, λ_k, (y-axis) for each of 20 countries (x-axis) in ten simulated data sets (panels).** Black dots and vertical lines denote posterior median and 95% credible intervals. Vertical red lines represent the 95% intervals of the prior distributions, sourced from a previous study [1]. Triangles correspond to the values of λ_k used to generate the simulated data.

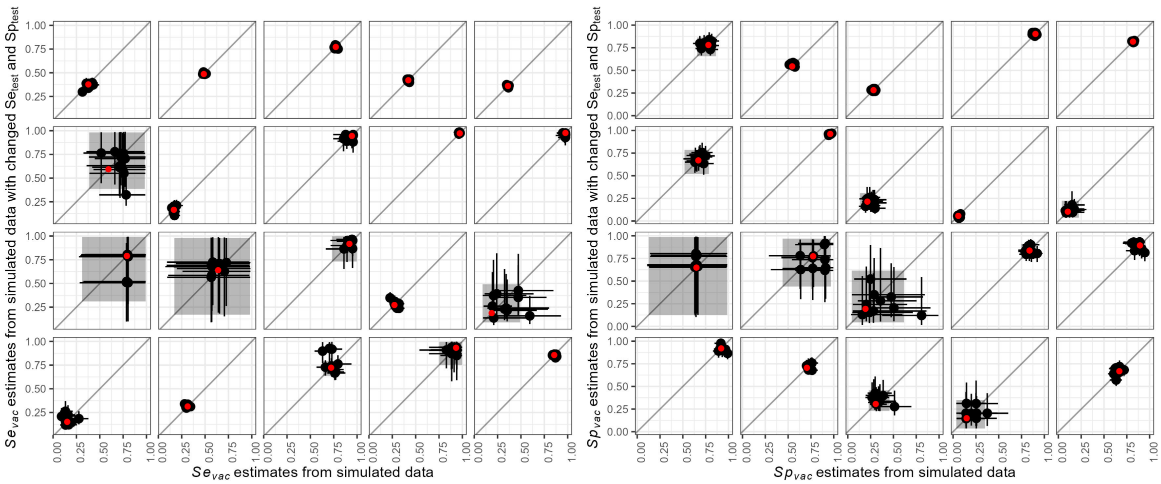

Figure S13: **Estimates of $Se_{vac,k}$ (left) and $Sp_{vac,k}$ (right) from simulated data with (y-axis) or without (x-axis) changed values of $Se_{test,k}$ and $Sp_{vac,k}$ for each of 20 countries (panels).** In the unchanged scenario, we simulated ten data sets with posterior median parameter values. In the changed scenario, we simulated ten data sets with $Se_{test,k}$ and $Sp_{test,k}$ set to 0.7. The ten black dots in each panel and their lines represent median estimates and 95% credible intervals obtained from our analysis of the ten simulated data sets. The red dots and gray areas represent median posterior estimates and their corresponding 95% credible intervals obtained from our analysis of empirical data.

2 Table

Table S1: **Correlation between $Se_{vac,k}$ and $Sp_{vac,k}$ across countries in eight models that account for geographic variability in Se_{vac} and Sp_{vac}.**

Se_{test} and Sp_{test}	FOI	Reporting proportion	Correlation
country	country	country	–0.852 (–0.924 - –0.711)
country	country	global	–0.853 (–0.926 - –0.720)
country	global	country	–0.854 (–0.925 - –0.726)
country	global	global	–0.855 (–0.922 - –0.728)
global	country	country	–0.855 (–0.925 - –0.709)
global	country	global	–0.854 (–0.924 - –0.718)
global	global	country	–0.856 (–0.925 - –0.727)
global	global	global	–0.857 (–0.923 - –0.726)

References

[1] T. Alex Perkins, John H. Huber, Quan M. Tran, Rachel J. Oidtman, Magdalene K. Walters, Amir S. Siraj, and Sean M. Moore. Burden is in the eye of the beholder: Sensitivity of yellow fever disease burden estimates to modeling assumptions. *Science Advances*, 7(42):eabg5033, October 2021. Publisher: American Association for the Advancement of Science.
